# A novel stepwise approach incorporating ethanol infusion in the vein of Marshall for the ablation of persistent atrial fibrillation

**DOI:** 10.3389/fcvm.2023.1194687

**Published:** 2023-05-16

**Authors:** Vasileios Sousonis, Stéphane Combes, Pauline Pinon, Nicolas Combes, Christelle Cardin, Sarah Zeriouh, Roberto Menè, Sophie Jacob, Serge Boveda, Jean Paul Albenque

**Affiliations:** ^1^ Heart Rhythm Management Department, Clinique Pasteur, Toulouse, France; ^2^ Institute for Radiological Protection and Nuclear Safety (IRSN), Fontenay-aux-Roses, France

**Keywords:** atrial fibrillation, persistent, ablation, ethanol, vein of Marshall

## Abstract

**Introduction:**

Apart from pulmonary vein isolation (PVI), several step-by-step procedures that aim to modify left atrial substrate have been proposed for the ablation of persistent atrial fibrillation (AF), yet the optimal strategy remains elusive. There are cumulative data suggesting an incremental benefit of adding vein of Marshall (VOM) ethanol infusion to PVI in patients with persistent AF. We sought to evaluate the feasibility and efficacy of a novel stepwise ablation approach, incorporating a VOM alcoholization step, for persistent AF.

**Methods:**

In this single-center study, we prospectively enrolled 66 consecutive patients with symptomatic persistent AF and failure of at least one antiarrhythmic drug (ADD). The ablation procedure consisted of (i) PVI, (ii) left atrial segmentation with VOM ethanol infusion and the deployment of linear radiofrequency lesions across the roof and the mitral isthmus and (iii) electrogram-based ablation of dispersion zones. The first two steps were performed in all patients, whereas the third step was carried out only in those still in AF at the end of the second step. Atrial tachycardias during the procedure were mapped and ablated. At the end of the procedure, cavotricuspid isthmus ablation was additionally performed in all patients. The primary endpoint was 12-month freedom from AF and atrial tachycardia after a single procedure and an initial three-month blanking period.

**Results:**

Total procedure time was 153 ± 38.5 min. Fluoroscopy time was 16 ± 6.5 min and the radiofrequency ablation time was 26.14 ± 0.26 min. The primary endpoint occurred in 54 patients (82%). At 12 months, 65% of patients were off any AAD. In the univariate Cox regression analysis, left ventricular ejection fraction < 40% was the only predictor of arrhythmia recurrence (HR 3.56; 95% CI, 1.04–12.19; *p* = 0.04). One patient developed a pericardial tamponade and another a minor groin hematoma.

**Conclusion:**

A novel stepwise approach, including a step of ethanol infusion in the VOM, is feasible, safe and provides a high rate of sinus rhythm maintenance at 12 months in patients with persistent AF.

## 1. Introduction

Catheter ablation for persistent atrial fibrillation (AF) is challenging and an optimal strategy has yet to be defined (1). Atrial ectopy arising from the pulmonary veins (PV) remains the main arrhythmia trigger in patients with persistent AF (2), yet the efficacy of pulmonary vein isolation (PVI) alone, in maintaining sinus rhythm, is modest (3). This exact fact highlights the importance of atrial myocardium in the pathophysiology of persistent AF, as it can be not only a source of non-PV triggers, but also a substrate favoring arrhythmia perpetuation.

Thus far, catheter ablation of complex fractionated atrial electrograms (CFAE) and atrial segmentation have been used as an adjunct to PVI, in an attempt to target atrial substrate in patients with persistent AF. The first approach refers to the ablation of areas demonstrating electrograms with spatiotemporal dispersion, which are believed to represent AF drivers and zones of continuous wavelet reentry (4, 5). Atrial segmentation is based on the Maze surgical procedure (6) and involves the creation of linear lesions that reduce excitable atrial mass and interrupt reentry circuits (7). Stepwise approaches combine multiple techniques to target areas that are critical for the initiation and perpetuation of persistent AF in a sequential manner, aiming at procedural sinus rhythm restoration, either spontaneously or by conversion of AF to an atrial tachycardia (AT), which is then mapped and ablated. The steps of these procedures typically consist of PVI and other thoracic veins isolation, ablation of areas demonstrating CFAE and, finally, left atrial (LA) segmentation, in case AF has not been terminated (8). This strategy, however, is associated with moderate long-term outcomes and extensive LA ablation that predisposes to arrhythmia recurrences and may require several repeat procedures to maintain sinus rhythm (9, 10).

The ligament of Marshall is known to promote arrhythmogenesis in patients with persistent AF, by serving both as a trigger and a substrate for local reentry (11). In addition, the ligament of Marshall accounts for arrhythmia recurrences after AF ablation (12). Ablation of this important structure can be effectively performed by ethanol infusion in the vein of Marshall (VOM) (13). When added to PVI, ethanol infusion in the VOM has been shown to improve long-term maintenance of sinus rhythm in patients with persistent AF undergoing catheter ablation (14).

In this prospective study, we investigated the feasibility and long-term outcomes of a novel stepwise ablation procedure incorporating a step of ethanol infusion in the VOM for patients with persistent AF. Our approach consisted of the following progressive steps: (i) wide antral PVI, (ii) VOM ethanol infusion and atrial segmentation with linear lesions at the mitral isthmus and the roof of the left atrium, and (iii) focal ablation targeting areas of electrogram dispersion in the left atrium.

## 2. Materials and methods

### 2.1. Study population

From November 2019 to June 2020, patients between 18 and 80 years old with symptomatic persistent AF and failure of at least one antiarrhythmic drug (AAD) were prospectively enrolled in the study. Exclusion criteria included: patients undergoing redo AF ablation, the presence of LA thrombus in preoperative imaging, the absence of the VOM, dissection of the coronary sinus (CS) and inability to administer ethanol in the VOM. The study was approved by the Institutional Review Board of our center and conformed to the ethical guidelines of the Declaration of Helsinki. All patients provided a written informed consent prior to the procedure.

### 2.2. Ablation procedure

All patients received oral anticoagulants for at least 30 days before the ablation. Transesophageal echocardiography or a cardiac computer tomography (CT) scan was performed within 48 h of the procedure to rule out atrial thrombi. Anticoagulation therapy was continued without interruption during the perioperative period. Heparin and heparinized saline were infused to maintain an activated clotting time of around 300 s. All procedures were carried out under general anesthesia.

Two experienced operators, both familiar with the techniques of this novel approach, performed all ablation procedures. A steerable quadripolar (Inquiry™, Abbott, St. Paul, MN, USA) or decapolar (Webster™, Biosense Webster, Diamond Bar, CA, USA) catheter was placed into the coronary sinus (CS). Access to the left atrium was obtained by a single transseptal puncture under fluoroscopic monitoring, using a fixed curve sheath (Swartz™ SL0, Abbot, St. Paul, MN, USA). High-density three-dimensional electroanatomic mapping was performed with a PentaRay™ multipolar catheter (Biosense Webster, Diamond Bar, CA, USA), using the CARTO 3® mapping system (Biosense Webster, Diamond Bar, CA, USA) and cardiac CT scan integration. The CartoUnivu™ module (Biosense Webster, Diamond Bar, CA, USA) was used to combine three-dimensional electroanatomic maps and fluoroscopy images during the procedures.

**Figure 1:**
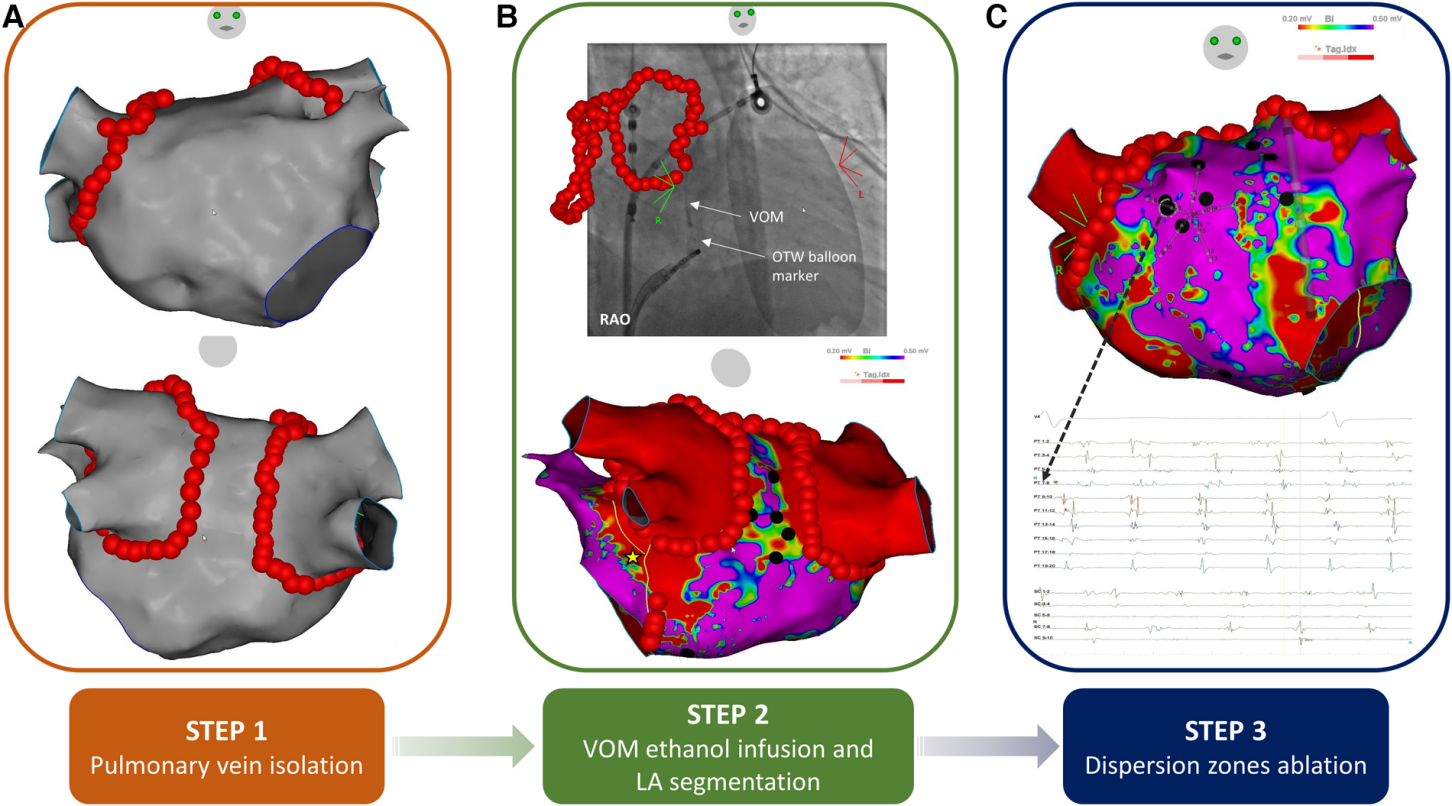
Schematic outline illustrating the three steps of the study ablation procedure. (**A**) Step 1: wide antral circumferential pulmonary vein isolation. (**B**) Step 2: VOM ethanol infusion and LA segmentation, upper panel: VOM venogram following vein occlusion with an over-the-wire angioplasty balloon; lower panel: LA voltage map after VOM alcoholization. The yellow line (star) represents the course of the VOM. A low-voltage area can be seen along the mitral isthmus and at the ridge between the LA appendage and the left pulmonary veins. LA segmentation was completed by additional RF applications to complete the mitral isthmus line and with the deployment of a roof line. (**C**) Step 3: Dispersion zones ablation, upper panel: LA voltage map with dispersion electrogram areas tagged with black dots; lower panel: example of dispersion electrograms. LA, left atrial; OTW, over-the-wire; RAO, right anterior oblique; RF, radiofrequency; VOM, vein of Marshall.

Sequential stepwise ablation was performed in all patients in the following steps: (i) radiofrequency (RF) wide antral circumferential PVI, (ii) ethanol infusion in the VOM and atrial segmentation with the deployment of linear RF lesions at the LA roof and the mitral isthmus (additional epicardial ablation through the CS was performed to achieve mitral isthmus block, if needed) and (iii) focal RF ablation of spatiotemporal dispersion zones (Figure 1). Regardless of AF termination, the procedure was carried on till the end of the second step in all patients, to ensure complete atrial segmentation. If AF persisted at the end of the second step, electrogram-based ablation of dispersion zones was performed with the endpoint of sinus rhythm restoration. If AF persisted at the end of the third step, electrical cardioversion was performed to restore sinus rhythm. In case AF evolved into an AT during any of the above steps, conventional AT mapping and ablation was performed. At the end of the procedure, RF ablation of the cavotricuspid isthmus was carried out in all patients, if not previously performed as part of the ablation of an intermediate AT. After restoration of sinus rhythm, entry and exit block was assessed at the PV antra and bidirectional block at every ablation line. In case of incomplete lesions, additional RF applications were administered.

Point-by-point RF ablation was performed in a power-control mode using an irrigated ablation catheter (ThermoCool SmartTouch® or QDOT micro®, Biosense Webster, Diamond Bar, CA, USA) at an irrigation rate of 4 to 20 ml/min. Maximal power was set at 50 W (25 W for ablation inside the CS) and the temperature was limited to 45°C. An ablation index of 400 was targeted at the posterior wall and the roof line, while for the anterior wall and the mitral isthmus line an ablation index of 500 was used. Temperature fluctuations in the esophagus were continuously monitored by a thermal probe (SensiTherm™, Abbott, St. Paul, MN, USA) and, in case temperature exceeded 40°C, energy application was discontinued.

Ethanol infusion in the VOM was performed as previously described with minor modifications (13). In brief, CS was cannulated with a fixed curve (Swartz™ SL0, Abbot, St. Paul, MN) or a deflectable (Agilis™, Abbot, St Paul, MN) sheath. A left internal mammary artery guide catheter was then used for angiographic contrast injection and localization of the ostium of the VOM, which was then sub-selectively cannulated. An angioplasty guidewire (Sion® blue, Asahi Intecc USA, Santa Anna, CA, USA) was advanced into the VOM and a pre-loaded over-the-wire angioplasty balloon, 1.5 or 2 mm in diameter (Sprinter™ OTW, Medtronic, Minneapolis, MN, USA) was then advanced into the proximal VOM and inflated. VOM anatomy and occlusion were confirmed by a venogram. Afterwards, 5 to 6 ml of ethanol (100%) were injected slowly through the central balloon lumen and the balloon was deflated.

When AF persisted after the first two steps, focal electrogram-based ablation targeting dispersion zones in the left atrium was performed. Electrogram dispersion areas were defined as previously described by Seitz et al. (5).

### 2.3. Patient follow-up

AADs were continued for 30 to 90 days after the ablation procedure. All patients were followed-up with clinical visits and 48-h Holter monitoring at 3, 6, and 12 months. During the inter-visit intervals, additional ECGs or Holter monitoring were performed in case of symptoms suggestive of atrial arrhythmias. The primary endpoint was freedom from any asymptomatic or symptomatic atrial tachyarrhythmia lasting more than 30 s, after a single procedure and an initial 3-month blanking period. Repeated ablation was offered to patients with arrhythmia recurrences.

### 2.4. Statistical analysis

Continuous variables are expressed as mean ± standard deviation and categorical variables as count and percentages. A Kaplan–Meier curve analysis was used to examine arrhythmia-free survival. Univariate and multivariate Cox proportional hazards regression models were used to assess factors associated with arrhythmia-free survival. For each variable, hazard ratios (HR) with corresponding 95% confidence intervals (CI) are reported. Statistical significance was established at *p* < 0.05.

## 3. Results

### 3.1. Patient characteristics

Sixty-six consecutive patients were enrolled in the study. Baseline characteristics are presented in Table 1. Mean age was 66 ± 9 years and 50 of the participants (76%) were males. All patients were in atrial fibrillation at the beginning of the procedure and mean AF duration of the current episode was 4.9 ± 3 months. All patients had at least one ineffective electrical cardioversion. Mean CHA_2_DS_2_-VASc score was 3 ± 1.2.

**Table 1:** Baseline characteristics (*n* = 66).

Clinical characteristics	
Age (years)	66 ± 9
Male gender	50 (76%)
BMI (kg /m^2^)	28.4 ± 4.6
Structural heart disease	18 (27%)
Heart failure	10 (15%)
Hypertension	34 (52%)
Sleep apnea syndrome	16 (24%)
CHA_2_DS_2_-VASc score	3 ± 1.2
Duration of current AF episode (months)	4.9 ± 3
History of cardioversion	66 (100%)
LVEF (%)	54 ± 12.7
LA volume (ml/m²)	56.5 ± 17.9
LA surface (cm^2^)	29 ± 5.4
AF appendage cycle length (ms)	146 ± 21

Data are presented as mean ± standard deviation or as count (%).

AF, atrial fibrillation; BMI, body mass index; LA, left atrial; LVEF, left ventricular ejection fraction.

### 3.2. Acute procedural outcomes

**Figure 2:**
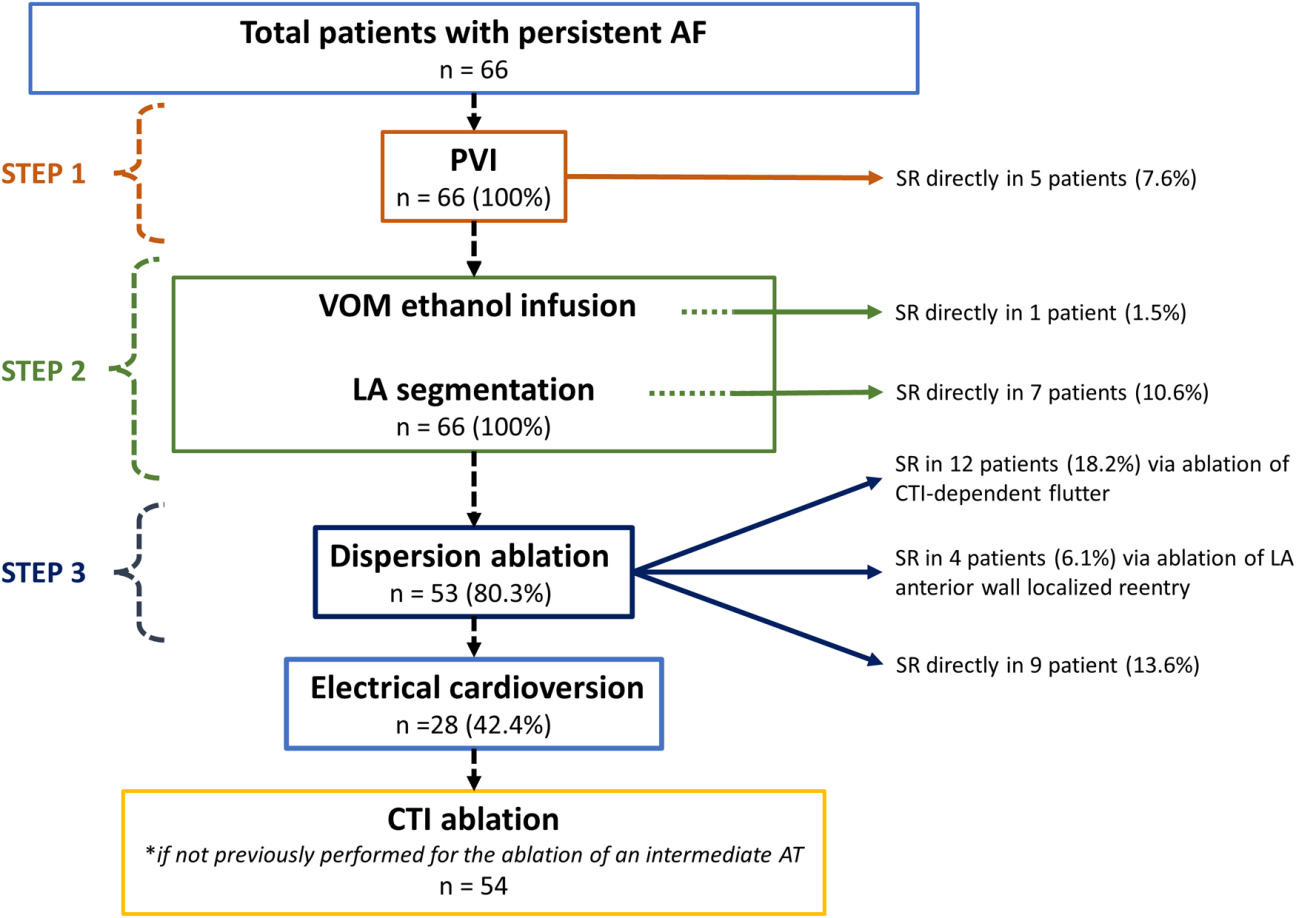
Study flow chart. AF, atrial fibrillation; AT, atrial tachycardia; CTI, cavotricuspid isthmus; LA, left atrial; PVI, pulmonary vein isolation; SR, sinus rhythm; VOM, vein of Marshall.

Total procedure time of the novel stepwise ablation strategy was 153 ± 38.5 min. Total fluoroscopy and RF ablation time was 16 ± 6.5 and 26.14 ± 0.26 min, respectively. Acute procedural outcomes are summarized in Figure 2. During the procedure, AF terminated directly to sinus rhythm in 22 patients (33.3%) and was converted to an intermediate AT in 16 patients (24.2%). Twenty-eight patients (42.4%) required electrical cardioversion at the end of the procedure to restore sinus rhythm. PVI was achieved in all patients and AF terminated to sinus rhythm solely by PVI in 5 patients. Thirteen patients did not have dispersion-targeted ablation, since sinus rhythm had already been restored prior to this final step. Bidirectional block at the mitral isthmus was achieved in 56 patients (85%), at the LA roof in 59 patients (89%) and at the cavotricuspid isthmus in 63 patients (95%). One patient developed cardiac tamponade requiring pericardiocentesis. Another patient experienced a groin hematoma, which was managed conservatively.

### 3.3. Follow-up

**Figure 3:**
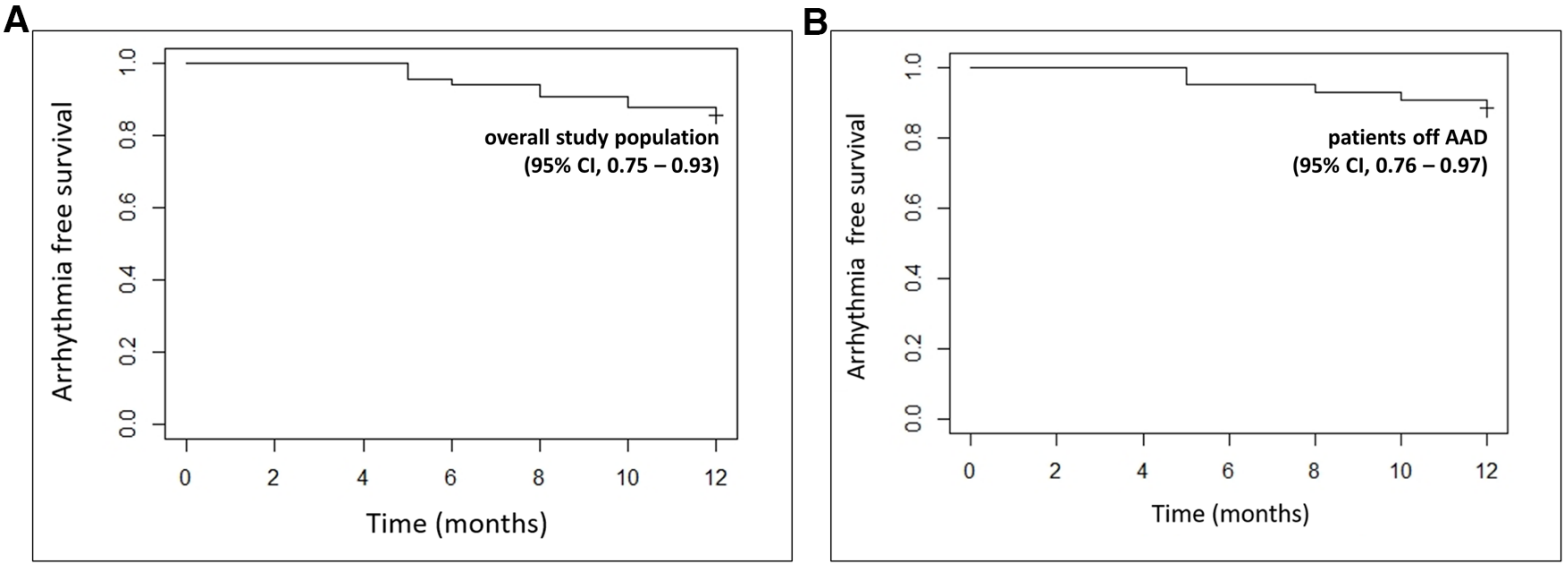
Kaplan-Meier arrhythmia-free survival curves after a single ablation procedure for the total study population (**A**) and for patients not taking any ADD. (**B**) 12-month 95% confidence intervals are provided in brackets. AAD, antiarrhythmic drug; CI, confidence interval.

After a single procedure, 54 patients (82%) remained free from AF or AT recurrences at 12 months. Five patients had recurrent persistent AF, while 7 patients presented with a macro re-entrant AT (5 patients with a perimitral and 2 patients with a roof-dependent LA flutter). At the end of the follow-up period, 43 patients (65%) no longer required an AAD. Among those taking AADs, 12 patients were on amiodarone, 7 patients on sotalol and 4 patients on flecainide. Kaplan Meier arrhythmia-free survival curves for the overall study population and for patients not taking any ADD are presented in Figure 3.

The results of the univariate and multivariate Cox proportional hazards regression analyses are summarized in Table 2. Left ventricular ejection fraction less than 40% was the only univariate predictor for arrhythmia recurrence during the follow-up period (HR, 3.56; 95% CI, 1.04–12.19; *p* = 0.04). Procedural AF termination was not found to have any influence on long-term sinus rhythm maintenance (HR, 0.68; 95% CI, 0.21–2.22; *p* = 0.52).

**Table 2:** Univariate and multivariate Cox proportional hazards regression analyses of factors affecting arrhythmia-free survival.

Baseline variable	Univariate HR (95% CI)	*p* value	Multivariate HR (95% CI)	*p* value
Age	1.02 (0.94–1.09)	0.67		
Sex (male)	1.38 (0.30–6.40)	0.68		
BMI	1.04 (0.92–1.16)	0.55		
Hypertension	0.48 (0.14–1.65)	0.24	0.60 (0.11–3.34)	0.55
CAD	1.57 (0.34–7.28)	0.56		
Sleep apnea syndrome	1.60 (0.47–5.47)	0.45		
LVEF < 40%	3.56 (1.04–12.19)	0.04	2.55 (0.36–17.84)	0.35
Duration of current AF episode	0.76 (0.52–1.11)	0.15	0.93 (0.36–1.36)	0.69
LA volume	1.02 (0.99–1.06)	0.25	0.99 (0.95–1.04)	0.77
Procedural AF termination	0.68 (0.21–2.22)	0.52		

AF, atrial fibrillation; BMI, body mass index; CAD, coronary artery disease; CI, confidence interval; HR, hazard ratio; LA, left atrial; LVEF, left ventricular ejection fraction.

## 4. Discussion

The key findings of our study can be summarized as follows: (i) addition of VOM alcoholization to a stepwise ablation procedure for persistent AF is feasible without raising any safety concerns and (ii) this novel ablation strategy is associated with high rates of freedom from AF and AT at one year follow-up.

Although PVI remains the cornerstone of AF ablation (15), the rates of a single ablation procedure in maintaining sinus rhythm in patients with persistent AF are as low as 57% (3). These results have led to a paradigm shift towards additional substate modification in patients with persistent AF. Multiple stepwise approaches have been proposed, most of them combining PVI, focal CFAE ablation and atrial segmentation in an attempt to increase procedural success. In 2005, Haïssaguerre et al. reported a 95% rate of medium to long-term sinus rhythm maintenance after a stepwise procedure (16). However, this was achieved at the cost of multiple procedures, since 38% of patients in this cohort required one or two repeat ablations due to recurrent arrhythmias, mainly AT. A meta-analysis in 2017 reported a 33% single- and 67% multiple-procedure success rate in maintaining sinus rhythm over a mean follow-up period of 2 years, when PVI and substrate ablation consisting of CFAE ± linear ablation were used in a step-by-step approach (3). Studies investigating a stepwise combination of PVI and CFAE ablation, with linear lesions being deployed only in case of AF conversion to an AT, reported an arrhythmia-free survival rate of around 57% at 12 months, after a single procedure (17, 18). The addition of a roof line to the ablation procedure has been reported to increase this rate to 69% (19). By incorporating a VOM ethanol infusion step and ensuring LA segmentation in all patients, we report a 82% arrhythmia-free survival rate at 12 months, after a single ablation procedure, in patients with persistent AF.

Cumulative evidence suggests that VOM ethanol infusion yields favorable outcomes, when added to an ablation procedure for persistent AF. The VENUS trial demonstrated a significant reduction in atrial arrhythmias at 12 months after a single procedure, without the use of AADs, in patients with persistent AF treated with VOM alcoholization in addition to PVI, as compared to those treated with PVI alone (14). Additional substrate modification was commonly performed at the discretion of the operator in both groups. The observed incremental benefit of VOM alcoholization on arrhythmia recurrences should be attributed to the arrhythmogenic potential of the ligament of Marshall. The ligament of Marshall is a structure that can exhibit early afterdepolarization-induced triggered activity and localized micro-reentry and may also serve as a component of macro-reentry circuits, as it carries various epicardial connections to the left atrium (20). The latter have been increasingly recognized as an important factor in the development of post-AF ablation AT (12, 21). Interestingly, VOM ethanol infusion results in durable lesions at the level of the mitral isthmus (22), where epicardial connections may render bidirectional block challenging, even after epicardial ablation through the CS. A *post hoc* analysis of the VENUS trial found that freedom of arrhythmia at follow-up was more likely when a complete mitral isthmus block had been achieved during the index procedure (23). With the novel stepwise procedure described in our study, we were able to achieve bidirectional mitral isthmus block in 85% of treated patients, a rate similar to those reported in the as-treated analysis of the VENUS trial (14) and the study by Laredo et al. (22). Regarding procedural AF termination, sinus rhythm was restored during VOM ethanol infusion in only one patient, in the present study. This may imply that the ligament of Marshall acts mainly as an AF trigger, rather than a substrate for arrhythmia perpetuation. Such a conclusion, however, cannot be safely drawn, since AF termination in stepwise procedures is the result of the cumulative effect of the structures targeted for ablation and the efficacy of an individual structure in restoring sinus rhythm depends on its position in the overall ablation sequence (24). Thus, the exact role of the ligament of Marshall in the complex pathophysiology of persistent AF merits further investigation.

Given the positive outcomes related to VOM alcoholization, other studies have also incorporated a VOM ethanol infusion step into sequential ablation procedures for persistent AF. In a prospective study, Derval et al. reported a 79% 12-month arrhythmia-free survival rate with no AADs in patients successfully undergoing a single index procedure, consisting of VOM ethanol infusion along with endocardial and epicardial RF ablation of Marshall bundle connections, wide antral PVI and linear lesions across the LA roof, the mitral and the cavotricuspid isthmus, an approach they called the Marshall-PLAN (25). Lai et al. found a 88.7% freedom from arrhythmia rate at 1 year in patients successfully treated with VOM ethanol infusion, followed by PVI and LA segmentation with a roof line, mitral and cavotricuspid isthmus ablation (26). Our results are in line with the above reported rates. Contrary to the aforementioned studies, though, we sought to combine an anatomical approach consisting of LA segmentation and VOM ethanol infusion in all patients with a final functional step targeting dispersion electrogram zones in patients not returning to sinus rhythm, as the latter may act as AF drivers (5). Since PVI and linear ablation are known to diminish CFAE zones (27), focal ablation of dispersion zones was performed as the last step in our approach. Moreover, in this way, we avoided extensive LA ablation that could predispose to arrhythmia recurrences (28).

When evaluating a new ablation strategy, extended follow-up periods are essential, since arrhythmia recurrences tend to increase with time (9). Clinical follow-up of our study was limited to a period of 12 months. Even so, important insights can be drawn from the comparative study of Liu et al. (29), in which a combination similar to that in our study, consisting of PVI, CFAE ablation and non-PV ectopy elimination, along with VOM alcoholization and linear lesions in case of rhythm transformation to a LA flutter (which were eventually performed in 84% of the patients) was tested. This approach was found to be quite efficient at 3.9 ± 0.5 years of follow-up, with the authors reporting a total atrial arrhythmia recurrence rate of 28%.

Widespread adoption of our approach could be hindered by the need for training on the VOM alcoholization procedure. This step requires dedicated materials and techniques that many electrophysiologists may not be familiar with. Nonetheless, VOM ethanol infusion early success rates range from 76% in lower-volume to 84% in high-volume centers, with subsequent improvement (30, 31). The two operators involved in our study were experienced in this technique, having performed more than 200 VOM alcoholization procedures in total, prior to the study. Collaboration with interventional cardiologists, familiar with basic angioplasty techniques, can help shorten the learning curve of this step.

## 5. Limitations

Specific limitations of our study should be acknowledged. First, this is a non-randomized study with a small sample size. As such, it should be viewed as a pilot study, the results of which need further validation in a larger randomized clinical trial. Additionally, short duration subclinical arrhythmia recurrences could not be ruled out, since our patients were not implanted with a loop recorder. However, following AF ablation, recurrences typically present as persistent, symptomatic arrhythmias that are more likely to prompt patients to seek medical consultation.

## 6. Conclusion

A novel stepwise ablation approach incorporating VOM ethanol infusion is safe and feasible, providing a high rate (>80%) of freedom from atrial arrhythmia recurrences at 12 months, in patients with persistent AF. This promising new strategy for persistent AF patients must be validated in a larger randomized study.

## Data Availability

The raw data supporting the conclusions of this article will be made available by the authors, without undue reservation.
